# Integration of single-cell datasets reveals novel transcriptomic signatures of β-cells in human type 2 diabetes

**DOI:** 10.1093/nargab/lqaa097

**Published:** 2020-11-20

**Authors:** Emanuele Bosi, Lorella Marselli, Carmela De Luca, Mara Suleiman, Marta Tesi, Mark Ibberson, Decio L Eizirik, Miriam Cnop, Piero Marchetti

**Affiliations:** Department of Experimental and Clinical Medicine, Pancreatic Islets Laboratory, University of Pisa, Pisa, I-56124, Italy; Department of Experimental and Clinical Medicine, Pancreatic Islets Laboratory, University of Pisa, Pisa, I-56124, Italy; Department of Experimental and Clinical Medicine, Pancreatic Islets Laboratory, University of Pisa, Pisa, I-56124, Italy; Department of Experimental and Clinical Medicine, Pancreatic Islets Laboratory, University of Pisa, Pisa, I-56124, Italy; Department of Experimental and Clinical Medicine, Pancreatic Islets Laboratory, University of Pisa, Pisa, I-56124, Italy; Vital-IT Group, SIB Swiss Institute of Bioinformatics, University of Lausanne, Quartier Sorge, CH-1015 Lausanne, Switzerland; ULB Center for Diabetes Research, Université Libre de Bruxelles, Brussels, B-1070, Belgium; Indiana Biosciences Research Institute (IBRI), Indianapolis, IN 46202, USA; ULB Center for Diabetes Research, Université Libre de Bruxelles, Brussels, B-1070, Belgium; Division of Endocrinology, Erasmus Hospital, Université Libre de Bruxelles, Brussels, B-1070, Belgium; Department of Experimental and Clinical Medicine, Pancreatic Islets Laboratory, University of Pisa, Pisa, I-56124, Italy

## Abstract

Pancreatic islet β-cell failure is key to the onset and progression of type 2 diabetes (T2D). The advent of single-cell RNA sequencing (scRNA-seq) has opened the possibility to determine transcriptional signatures specifically relevant for T2D at the β-cell level. Yet, applications of this technique have been underwhelming, as three independent studies failed to show shared differentially expressed genes in T2D β-cells. We performed an integrative analysis of the available datasets from these studies to overcome confounding sources of variability and better highlight common T2D β-cell transcriptomic signatures. After removing low-quality transcriptomes, we retained 3046 single cells expressing 27 931 genes. Cells were integrated to attenuate dataset-specific biases, and clustered into cell type groups. In T2D β-cells (*n* = 801), we found 210 upregulated and 16 downregulated genes, identifying key pathways for T2D pathogenesis, including defective insulin secretion, SREBP signaling and oxidative stress. We also compared these results with previous data of human T2D β-cells from laser capture microdissection and diabetic rat islets, revealing shared β-cell genes. Overall, the present study encourages the pursuit of single β-cell RNA-seq analysis, preventing presently identified sources of variability, to identify transcriptomic changes associated with human T2D and underscores specific traits of dysfunctional β-cells across different models and techniques.

## Graphical Abstract

**Graphical Abstract:**
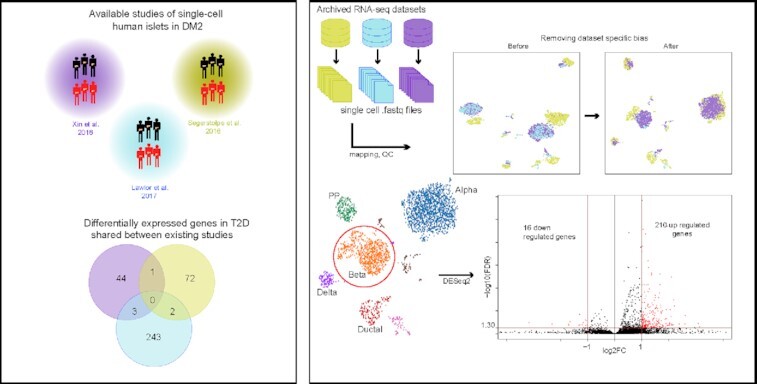
Available studies of human islets from type 2 doa and control donors revealed no shared differentially expressed genes (left). Their integrated analysis allowed to attenuate dataset-specific biases and highlight new signatures of beta-cell dysfunction (right).

## INTRODUCTION

The last decade showed a sharp increase in our ability to investigate whole transcriptomes at a high resolution. In parallel to the continuous improvements of sequencing platforms, the emergence of single-cell RNA sequencing (scRNA-seq) (1) made it possible to obtain transcript sequences out of individual cells, enabling to capture features of cellular differentiation, pathogenesis and adaptation (2–4), which would have been overlooked using bulk RNA-seq. The applications of such technology are very promising, especially for the study of heterogeneous tissues containing different cell types or the analysis of rare cells, in that it allows to characterize which genes are selectively expressed in different cell types, to reconstruct the trajectories of cell differentiation and response to stimuli (5,6) and to infer underlying regulatory networks (7). Altogether, scRNA-seq has the potential of filling knowledge gaps in our current understanding of how genetics and environmental factors affect the phenotype of single cells, and how these in turn influence the structure–function of tissues and organs (8).

The heterogeneous nature of pancreatic tissue makes it an excellent target to be analyzed with scRNA-seq. In fact, the organ is made up of a number of different cell types having either exocrine or endocrine secretory functions. Cells belonging to the latter category are found in the islets of Langerhans, which are cell clusters predominantly composed of α, β, δ and PP cells that secrete glucagon, insulin, somatostatin and pancreatic polypeptide, respectively. The β-cells are the sole source of insulin produced in the human body, and are therefore strictly implicated in the onset and progression of type 2 diabetes (T2D) (9,10). Therefore, the molecular and physiological characterization of β-cells in T2D is central for the identification of specific pathways and functions associated with their failure, which could provide novel insights into T2D pathophysiology for better prevention and treatment of this disease. Importantly, β-cells are probably heterogeneous (11,12), which may affect how putative β-cell subpopulations respond to the predisposing genetic background and metabolic stresses leading to T2D.

So far, scRNA-seq has been applied to human islets from non-diabetic (ND) and T2D donors in three valuable independent studies (13–15) in an effort to identify differentially expressed genes (DEGs) in T2D. A comparison of the sets of DEGs in β-cells from these studies revealed, surprisingly, that not a single gene was shared (16). This discrepancy could be due to the complex etiology of T2D and the (relatively) limited number of donors analyzed; it should also be considered that these studies had different experimental and analytical steps, from the isolation of single cells to the computational analysis of sequenced reads, which inevitably add technical sources of variability that can confound biologically relevant data (17,18).

The single-cell field is witnessing an incredibly fast progression, with the establishment of toolkits such as Scanpy (19) or Seurat (20) that enable the seamless implementation of standardized analytical workflows to scRNA-seq data. This, coupled with the definition of better guidelines and standards (21), not only makes it easier to integrate datasets within a single analytical design to correct for study-specific bias (22), but also removes the influence of technical biases arising from different computational tools and algorithms.

In this study, we aimed to deliver a comprehensive picture of the human pancreatic single β-cell transcriptomes in T2D. To do so, we integrated the three major scRNA-seq studies of human islets in a single dataset that was then analyzed by focusing on β-cells to identify shared DEGs and pathways to reconcile the identified features of T2D β-cells with the current biological knowledge of this condition (23–25). To evaluate the consistency of our findings, we also compared our results with those of (i) another study of β-cells from T2D patients and controls, based on an orthogonal methodology, namely laser capture microdissection (LCM) (26), and (ii) islets from a rat model of pancreatectomy-induced hyperglycemia (27).

## MATERIALS AND METHODS

### Analysis of sequencing data and dataset integration

The fastq files from the three studies re-analyzed in this work were downloaded using SRA toolkit (https://ncbi.github.io/sra-tools/) for the projects archived in SRA (28) (SRP075377 and SRP075970), or custom bash script for the one deposited in ArrayExpress (29) (E-MTAB-5061). Metadata reporting information for each cell, including donor ID, gender, body mass index and diabetic condition, were downloaded as well from the respective repositories. For one dataset (14), we excluded cells not having values of the quality metadata as ‘OK’.

The reads were aligned against the human reference genome GRCh37 (Ensembl 87 annotation) using STAR 2.7.3 (30) with ‘--quantMode TranscriptomeSAM GeneCounts’, obtaining for each study a table reporting per-gene read counts of each cell. From this point onward, all downstream analyses were conducted using ad-hoc Python scripts implementing functions from the toolbox Scanpy (19). The read count files were integrated with the cell metadata and the Ensembl annotation to produce three AnnData files, which were used to perform cell-wise quality control (QC) analyses.

Defining the number of read counts per sample as ‘counts’, the number of genes with at least one read mapped as ‘expressed genes’ and the ratio of reads mapped on mitochondrial genes as ‘mitochondrial fraction’, we considered counts, expressed genes and mitochondrial fraction as technical covariates defining the quality of each cell. Specifically, cells with relatively high number of counts and genes are likely representing multiplets, i.e. two or more cells captured and sequenced assuming a single cell, whereas high mitochondrial fraction and low expressed genes are indicative of lysed cells. We considered the distribution of these variables and their covariation separately for each dataset, defining separate threshold values that allowed to identify and flag cells as ‘low quality’, which were then excluded from downstream analysis. Contemporary with cell-level QC, genes expressed in less than three cells or expressed only in a single dataset were not considered for downstream analyses.

After QC, the datasets were concatenated and the counts were normalized by scaling the count values to obtain a total count of 10 000 for each cell and then log transformed. This normalized dataset was analyzed to compute the dispersion of each gene with respect to its mean value to annotate genes as highly variable using the ‘highly_variable_genes’ function of Scanpy. This allowed to define a set of 971 genes displaying a high variability in each single dataset, which was used to perform dataset integration with mutual nearest neighbors (MNN) algorithm (22) as implemented in mnnpy (https://github.com/chriscainx/mnnpy). Visualization of single cells before/after MNN correction was performed with uniform manifold approximation projection (UMAP) as implemented in Scanpy.

### Unsupervised clustering and cell type annotation

Detection of single-cell communities was done using the Louvain modularity algorithm (31) implemented in Scanpy (https://github.com/vtraag/louvain-igraph) with resolution = 0.5. The relative contribution of genes in separating clusters was computed with the ‘rank_genes_groups’ function of Scanpy, manually evaluating their association with major pancreatic cell types using literature information and gene expression markers reported in PanglaoDB (32).

### Differential expression in T2D and enrichment analyses

DEGs in T2D β-cells were identified using DESeq2 (33) with the following design: *Counts ∼ Dataset + Diabetes*, where *Counts* is the matrix of raw count data, *Dataset* is a three-level factor (SEG, XIN, LAW) indicating the dataset of origin and *Diabetes* is a two-level factor (T2D, ND) indicating the diabetes status of the donor. Genes were considered as DEG if passing these thresholds: <0.05 for corrected *P*-value (false discovery rate, FDR) and >2 for the absolute value of fold change (FC). DEG identification for individual datasets was performed similarly, with the following design: *Counts ∼ Diabetes*.

Gene set enrichment analysis (GSEA) was performed using Enrichr with the following datasets: the Gene Ontology 2018 (GO) subsets Biological Process, Molecular Function and Cellular Component (34,35); BioPlanet 2019 (36); KEGG 2019 (*Homo sapiens*) (37–39); Reactome 2016 (40); and a consensus of transcription factor target genes from Encode and ChEA. All gene sets are available at https://amp.pharm.mssm.edu/Enrichr/#stats.

Overlapping expression trends were identified with two-tailed rank–rank hypergeometric overlap (RRHO) (41,42) of genes ranked according to −log(*P*-value) *×direction*, where *direction* is the sign of the expression change in T2D versus ND. A custom R script implementing the RRHO package (v. 1.24.0) was used to compute and analyze the hypergeometric distribution matrix, which allowed us to identify genes corresponding to overlapping expression trends.

### Coding and data visualization

To visualize the obtained results, ad-hoc Python scripts were used combining Scipy (43), Pandas, Matplotlib (44), Seaborn and Scanpy (19). The volcano plot of DEGs was produced with R (https://www.r-project.org/). The analytical workflow was organized using a Jupyter notebook.

## RESULTS

### A merged scRNA-seq dataset of islets from T2D patients

**Figure 1.**
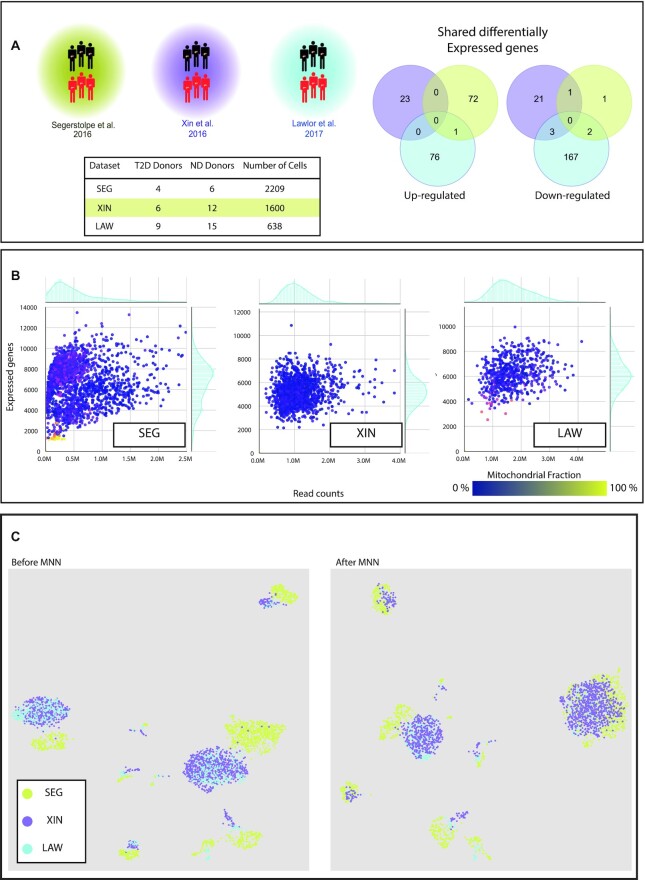
An integrated dataset of single-cell transcriptomes from studies of human islets (T2D versus ND). (**A**) The three studies considered in this work are Segerstolpe (SEG) (14), Xin (XIN) (13) and Lawlor (LAW) (15) that analyzed the indicated total number of cells from the indicated number of T2D and ND donors. The Venn diagram recapitulates the previous comparison of DEGs (by Wang and Kaestner) (16), showing paucity of shared DEGs. (**B**) The plots report the number of expressed genes, total read counts and the mitochondrial enriched fraction of the cells of each dataset. Extreme values of these parameters indicate low-quality cells, as cells with high read counts and expressed genes might represent multiplets of captured cells, while a high fraction of mitochondrial genes expression is indicative of lysed cells (21). The histograms on the axes of each plot represent the marginal distributions of the corresponding variables (i.e. read counts: top; expressed genes: right). (**C**) UMAP visualization of the integrated dataset before (left) and after (right) the normalization with MNN. Cells are colored according to the dataset of origin to highlight the effect of the MNN normalization.

We obtained raw sequencing data of three studies: E-MTAB-5061 (14), SRP075377 (13) and SRP075970 (15) (hereafter referred to as SEG, XIN and LAW, respectively) (Figure 1A), whose deposited sequences represent the transcriptome of single cells passing QC checks as defined by the respective authors. Read count matrices reporting the relative gene expression in each cell were also available, but we decided not to use them since different approaches were used to obtain them from raw data. By re-analyzing the original reads with the same pipeline, we removed variability coming from usage of different tools.

As shown in Table 1, the datasets differed in terms of donor selection, single-cell isolation and sequencing library preparation, but also in QC criteria for expressed genes, total read counts and mitochondrial fraction (Figure 1B). For the number of expressed genes, we found a unimodal distribution for XIN and LAW with a peak around 5500, whereas SEG showed a bimodal distribution with peaks around 4000 and 8000. We also found SEG to display a higher variance for total read counts, having a high number of cells with extremely low read counts and outliers with expression up to 6 million reads, and high mitochondrial fraction, with outliers having values close to 1. XIN had no cells with mitochondrial fraction >0.25, reflecting differences in QC criteria used in the original studies.

**Table 1.** Main features of the datasets used

Dataset	Number of donors (T2D, ND)	Number of cells	Read length	Average million read count (std)
SEG	10 (4, 6)	2209	43	0.558 (0.525)
XIN	18 (6, 12)	1600	75	1.145 (0.551)
LAW	24 (9, 15)	638	75	1.689 (0.653)

The table reports the different features of the datasets used, in terms of number of donors (total, T2D and ND), number of cells with available raw sequencing data, length of sequencing reads and the average number of reads (million) per cell.

We excluded cells with signatures of low viability (i.e. low count depth, high fraction of mitochondrial genes) or multiplets, defined as multiple cells sequenced and labeled as a single cell. We considered each dataset separately to define threshold values for metrics such as number of expressed genes, total read counts and fraction of read counts on mitochondrial genes (see Table 2). We also excluded outlier genes, defined as those expressed in less than three cells or being present in a single dataset.

**Table 2.** QC filtering criteria

Dataset	Number of cells before QC	Number of good quality cells	Number of genes before QC	Number of genes after filtering	Range of expressed genes	Range of total read count (million)	Maximum mitochondrial fraction
SEG	2209	1148	33 466	27 931	2500–9000	0.3–2	0.4
XIN	1600	1382	33 466	27 931	2500–7000	0.5–2.2	–
LAW	638	516	31 511	27 931	2500–9000	1–3	0.4

The table reports for each dataset the number of cells and genes before and after the QC filtering, as well as the threshold values used to identify good quality cells. Ranges are pairs of values indicating the lower and the upper bound thresholds.

After removing the cells not satisfying the QC criteria, we concatenated the data into a single merged dataset, containing (i) a gene count matrix embedding 3046 cells and 27 931 genes, and (ii) a metadata matrix with the ancillary information available for each cell and donor. The dataset is available as an h5ad file (see the ‘Data availability’ section).

### Normalization of dataset effects and cell-level analyses

Considering that our integrated dataset contains data from three laboratories using different protocols and technologies, gene expression might present systematic differences due to batch effects. In order to correct this technical source of variation, we applied a data integration method (MNN correction) on our dataset to better highlight biological features shared between cells. To visualize the effects of such correction, we identified a set of 971 highly variable genes, which were used for UMAP two-dimensional representation of the cells labeled according to the dataset of origin.

This visualization (Figure 1C) shows clearly separated groups of cells with similar transcriptomes, with LAW and XIN exhibiting remarkable overlap, whereas SEG is more distinct. After MNN correction (Figure 1C), there was a larger overlap of SEG with the other datasets, indicating a reduction of the dataset effect on the transcriptomic differences between cells.

**Figure 2.**
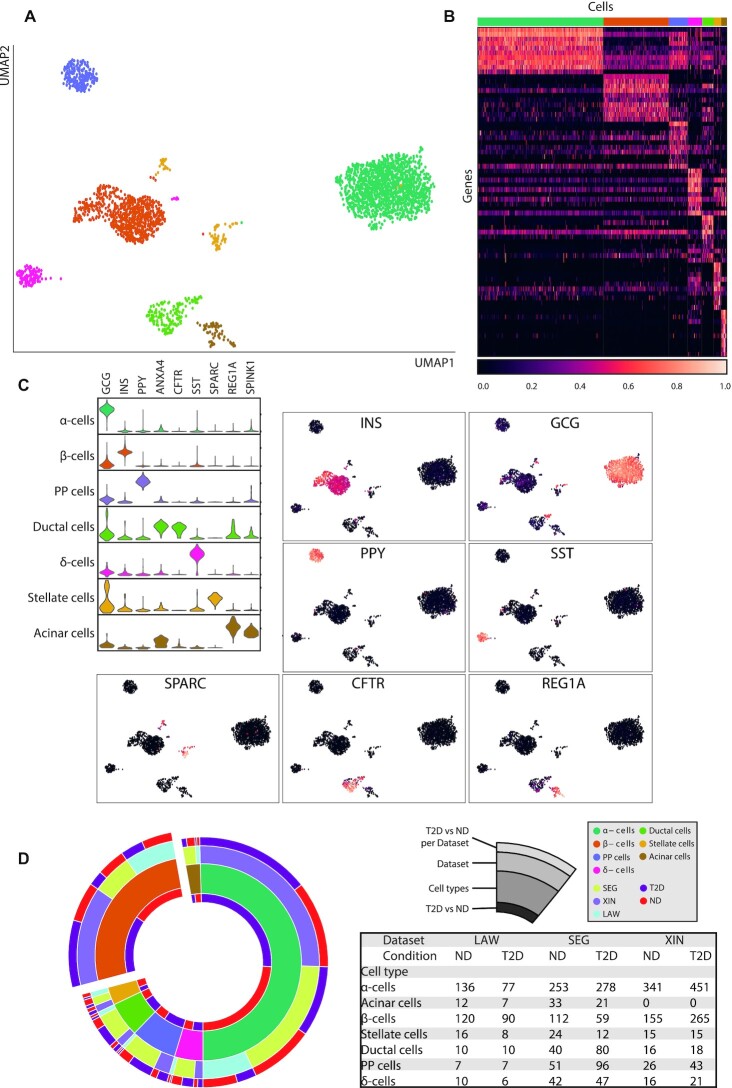
Analysis of the integrated dataset at the cell level. (**A**) The UMAP visualization of the integrated dataset (after MNN) with cells color labeled according to the clusters assigned with the unsupervised method Louvain (31). (**B**) The heatmap reports the normalized expression of the 10 most representative genes of each cluster (rows) in each cell (columns). Columns are color labeled according to cell clusters. (**C**) Distribution of the expression of marker genes for each cell type (violin plots). Normalized expression of the most representative marker genes in each cell with a UMAP visualization. (**D**) The sections in the circular plot represent the cell counts stratified in different categories. From outer to inner: donor diabetes condition (T2D, ND) per dataset and cell type, dataset (SEG, XIN, LAW), cell types and donor diabetes condition (T2D, ND) per cell type with aggregated datasets.

We next performed unsupervised clustering of the cells based on their corrected gene expression profiles, obtaining seven different cell clusters, i.e. α, β, δ, PP, ductal, stellate and acinar cells (Figure 2A). From the transcriptomic signatures of the identified groups, we selected genes maximizing the diversity between groups to annotate their identity (Figure 2B and Supplementary Figure S1). This led to the identification of nine marker genes (GCG, INS, PPY, SST, ANXA4, CFTR, SPARC, REG1A and SPINK1) whose expression is associated with specific pancreatic cell types (Figure 2C). This information allowed us to characterize the cell type composition of our dataset at different hierarchical resolution (aggregated, dataset and individual level) and related to disease state (T2D versus ND), testing the enrichment of cell types in each condition. We observed substantial differences in the abundance of cell types between different datasets (Figure 2D). SEG had half the proportion of β-cells of other datasets (15% versus 30% and 41%), and substantially higher ductal, δ and PP cells. Acinar cells were missing in XIN.

### Differentially expressed genes in T2D β-cells

A previous study comparing scRNA-seq transcriptomic signatures of T2D showed no shared DEGs (16). To assess how much of this variability is effectively due to differences in data processing, we identified DEGs separately for each dataset and compared them. Since no DEGs are shared between datasets (Supplementary Figure S2), variability observed is probably due to different sample source, preparation and other experimental steps rather than data processing.

**Figure 3.**
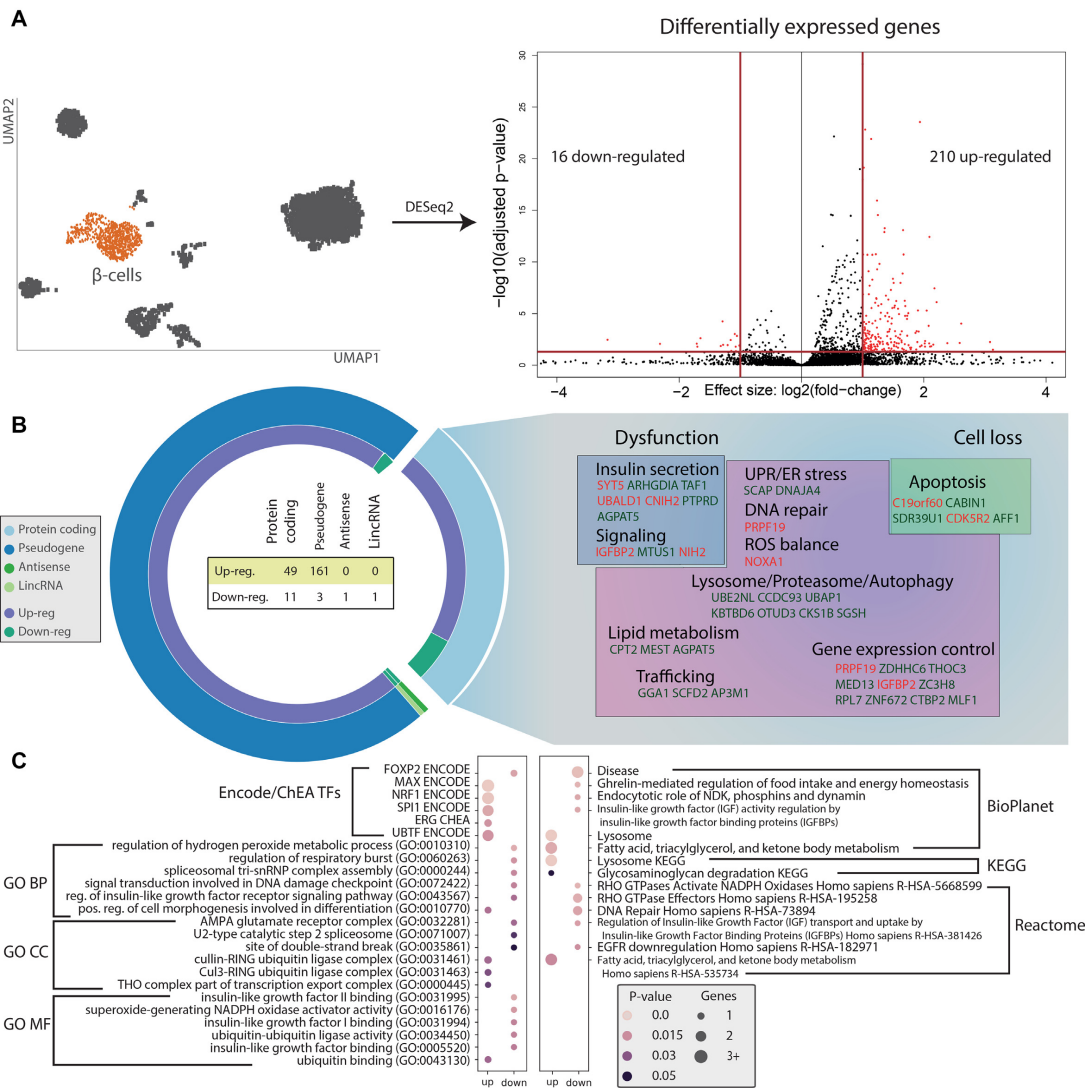
Analyses of the integrated dataset at the gene level. (**A**) Volcano plot reporting the significance and FC values obtained for each β-cell gene with DESeq2. The horizontal and vertical red lines report the threshold used to define DEGs (shown in red). (**B**) Classification of DEGs according to biotype (outer circle) and direction of change (inner circle). The gene symbols associated with T2D-related functions are reported colored differently according to the corresponding expression change direction (green: upregulated; red: downregulated). (**C**) Terms enriched in the DEG set. For each term, the number of associated genes (dot size) and enrichment significance (color scale) are reported. The reported terms correspond to the six most significant terms for each dataset.

Using our merged dataset, we regressed out bias due to the dataset of origin. Comparing the T2D versus ND β-cell subpopulations, we identified 226 DEGs (FDR ≤ 0.05, FC ≥ 2), with 210 upregulated and 16 downregulated genes in T2D (see Figure 3A). Of these, 60 were protein-coding genes (Figure 3B), which were manually curated based on the available literature and databases (i.e. UniProt, STRING, KEGG and GWASdb) to explore their pathophysiological role (Supplementary Table S1). For 35 DEGs, their function could be related to T2D, including β-cell failure mechanisms, such as defective insulin secretion, increased oxidative stress, altered autophagy and apoptosis (24,25,45–47) (Figure 3B). The remaining 25 genes have not been described previously. Of these, 16 genes have an undefined function, whereas the other 9 could be ascribed to cellular processes linked to β-cell dysfunctions (Supplementary Table S1). These include CABIN1, CKS1B, C19orf60 and SDR39U1 (affecting cell survival), SLC31A1 (involved in copper homeostasis), DNAJA4 (associated with ER stress), ZC3H8 (regulating the expression of GATA3), OTUD3 and UBALD1 (affecting ubiquitination) (48–58).

We then performed GSEA on seven datasets using Enrichr. The results of this analysis showed an enrichment of several categories that can be associated with altered β-cell pathways (Figure 3C and Supplementary Table S2), including the control of hydrogen peroxide and respiratory burst (GO:0010310, GO:0060263), the activity of NADPH oxidase for ROS generation (GO:0016176) and the ionotropic glutamate receptor (GO:0008328). Of interest, a pathway that appeared positively enriched in multiple datasets is related to lysosome function, which is associated with autophagy (59).

### Generalizing common transcriptomic signatures of T2D β-cells across different methodologies

To verify the extent to which our results may be generalizable, we compared the gene expression signatures of the present integrated transcriptomic dataset with those from two other models. We used (i) the results obtained by microarray gene expression analysis of human T2D and ND β-cell enriched samples yielded by LCM (60), as a model similar to the human single β-cell approach, and (ii) the data recently generated by islet RNA-seq assessment in 90% pancreatectomized, hyperglycemic rats (27), as a less close model.

**Figure 4.**
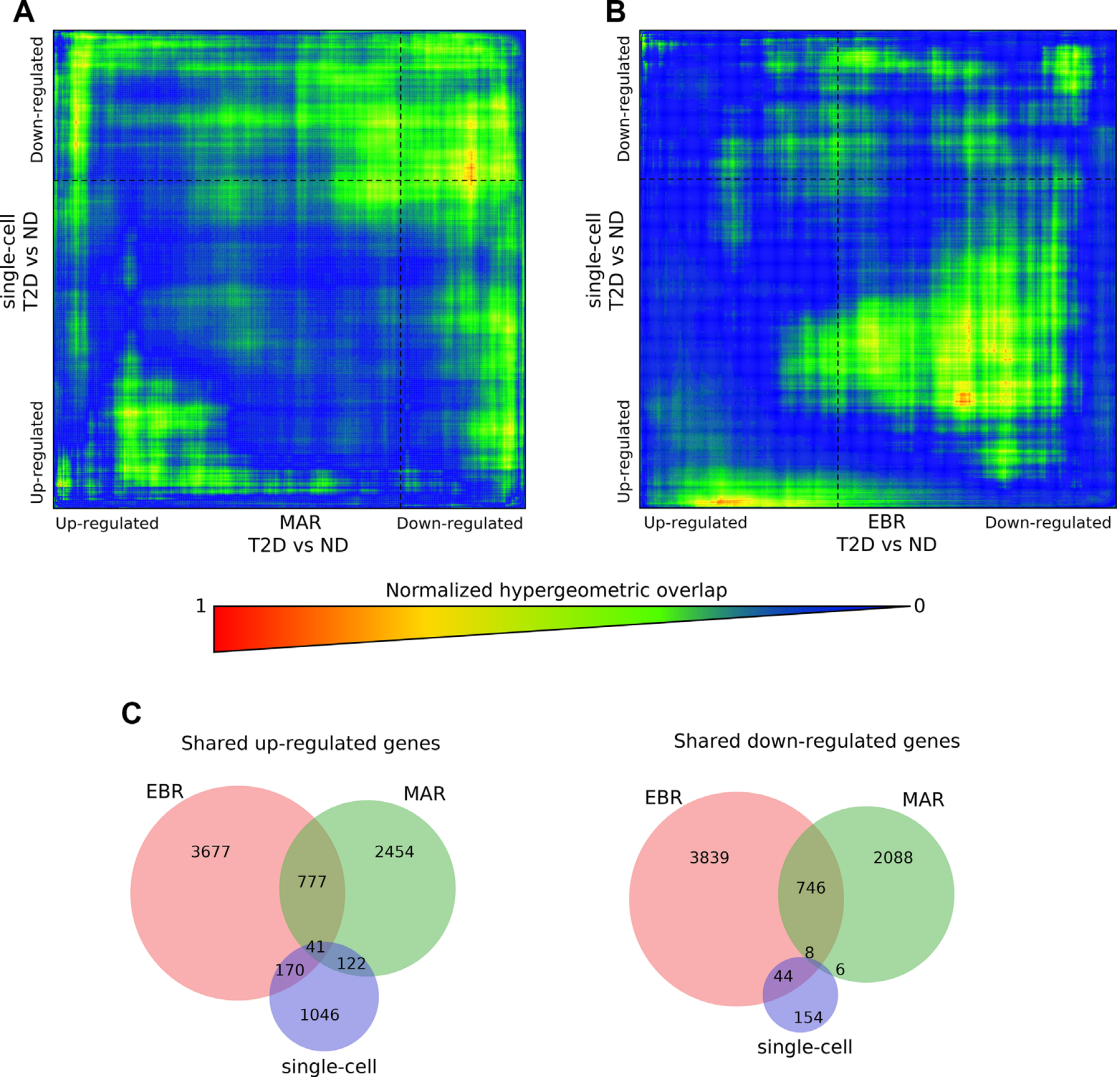
Shared transcriptomic patterns between single β-cells, MAR and EBR. (**A**) RRHO map showing overlap between the single-cell differential expression of T2D versus ND and the differential expression of T2D versus ND in MAR. A detailed description of the MAR dataset is provided in the text. Genes are ranked by FC from most downregulated to most upregulated. The level map colors show normalized −log *P*-values for overlap, with an indication of the smallest *P*-value for clusters with statistically significant overlap between genes upregulated in both datasets (bottom left quadrant), downregulated in both (top right quadrant), upregulated in MAR T2D and downregulated in single-cell T2D (top left quadrant), and downregulated in MAR T2D and upregulated in single-cell T2D (bottom right quadrant). (**B**) RRHO map showing overlap between the single-cell differential expression of T2D versus ND and the differential expression of T2D versus ND in EBR. A detailed description of the EBR dataset is provided in the text. Genes are ranked by FC from most downregulated to most upregulated. The level map colors show normalized −log *P*-values for overlap, with an indication of the smallest *P*-value for clusters with statistically significant overlap between genes upregulated in both datasets (bottom left quadrant), downregulated in both (top right quadrant), upregulated in EBR T2D and downregulated in single-cell T2D (top left quadrant), and downregulated in EBR T2D and upregulated in single-cell T2D (bottom right quadrant). (**C**) Differentially regulated genes shared in the present integrated single β-cell dataset, MAR and EBR data. The Venn diagrams indicate the number of DEGs (*P* ≤ 0.05) shared between the single-cell dataset, MAR and EBR. The diagrams separate upregulated (left) and downregulated (right) genes.

In a first set of analyses, we used the RRHO approach (41) that allows to compare differentially expressed transcriptomes between independent studies in a threshold-free way and visualize both the significance and direction of the possible overlays. In the work by Marselli *et al.* (26), hereafter referred to as MAR, the analysis of 10 T2D and 10 ND β-cell enriched samples identified 1742 DEGs utilizing a significance threshold of lower confidence bound ≥1.2, corresponding to 1086 upregulated and 656 downregulated genes. The comparison of the transcriptomes of MAR T2D versus ND with those of our integrated T2D versus ND single β-cell transcriptome assessment is reported in Figure 4A. The analysis revealed that although there were genes regulated in opposite direction, 191 transcription signatures overlapped between the two studies (173 upregulated and 18 downregulated). Among them, there were 20 genes with *P*-value ≤0.05, of which 11 have been previously described in association with T2D traits (61–71) (Supplementary Table S3).

In the study by Ebrahimi *et al.* (27), hereafter referred to as EBR, the authors characterized rat islet transcriptomic changes following 90% pancreatectomy. At 10 weeks after surgery, pancreatectomized, hyperglycemic animals showed many islet transcriptome changes in comparison with controls. There were 7844 DEGs, many of which associated with glucose toxicity, stress, inflammation and β-cell identity. The RRHO comparison of EBR data with the present single β-cell transcriptome results revealed that there were 1014 common genes that were upregulated (Figure 4B). Among them, 118 genes were significantly (*P* ≤ 0.05) regulated in both datasets (Supplementary Table S3), and 61 of these genes have been previously linked to T2D features (72–126) (Supplementary Table S3).

Next, we compared the three datasets (integrated single cells, MAR and EBR) to identify shared genes (Figure 4C). We considered only genes with *P* ≤ 0.05 and found a set of 208 genes, of which 41 and 8 were, respectively, upregulated and downregulated in all three datasets (Supplementary Table S4). Interestingly, several of such genes (20 upregulated and 4 downregulated) have been reported to be linked to diabetes (74,76,91–93,111,112,126–138) (Supplementary Table S4).

## DISCUSSION

The present work was prompted by the observation that, comparing the results of the three available studies of pancreatic β-cells from individuals affected by T2D and ND controls (13–15), scRNA-seq ‘failed’ to deliver a shared view of T2D-associated transcriptomic alterations of β-cells from human islets, possibly due to a number of methodological issues (16). Here, we show that an integrated analysis of the three datasets, based on recently developed algorithms and computational frameworks specific for single-cell transcriptomics (19,21,22), allowed us to identify genes that were differentially expressed in T2D versus ND β-cells, with potential pathophysiological roles.

These new tools mentioned above have made it possible to integrate different datasets and deal with technical covariates, allowing the re-analysis of published data to obtain novel biological insights (22). In particular, the pan-transcriptome we obtained by merging the published scRNA of human β-cells (13–15) has a larger sample size, hence is more robust against biases arising from donor-specific sources of biological variability (including a supposed effect of multiple and various T2D etiologies). For instance, this approach allowed us to reconcile discrepancies between the results of the three different studies. As an example, the genes TUBA1B and LEPROTL1 were downregulated in T2D according to LAW, upregulated according to SEG and not significantly different according to XIN; according to our analysis, these genes are not differentially expressed.

We strived to minimize technical sources of variabilities that could affect our analyses. Indeed, the comparison of QC metrics revealed a divergence between datasets at the cellular level that likely reflect separate selection criteria operated by the respective authors. Briefly, the original studies were not uniform concerning the minimal quality level of cells to be subjected to downstream analyses, with effects that include a different heterogeneity within cell types observed by the authors. For instance, Segerstolpe *et al.* (14) reported heterogeneity for the β-cell group, whereas Xin *et al.* (13) did not. To obtain cells with comparable quality levels and reduce this bias, we adopted a conservative approach using different thresholds for each dataset to remove low-quality cells. Even with such harmonization, UMAP visualization of transcriptomes revealed a separation of cells according to the dataset of origin, implying that differences in experimental procedures, from islet culturing to RNA-seq library preparation and sequencing, impact the islet cell transcriptomes. To minimize this effect, we used MNN (22,139), but standardized procedures and materials in future studies will increase reproducibility.

Our integrated dataset identified 226 DEGs in β-cells associated with T2D, most of which were overexpressed. Since we used the T2D condition to contrast the transcriptomes, we expected some DEGs to recapitulate known signatures of diabetes. Indeed, among the 60 differentially expressed protein-coding genes there were 35 genes with functions related to β-cell damage, such as impaired insulin secretion, increased oxidative stress, deranged autophagy and apoptosis (24,25,45–47) (Figure 3B). In addition, we identified nine genes (CABIN1, CKS1B, C19orf60, SDR39U1, SLC31A1, DNAJA4, ZC3H8, OTUD3 and UBALD1) not previously associated with diabetes, but that are known to be involved in processes potentially linked to β-cell dysfunction, such as cell turnover, oxidative stress and ER stress (Supplementary Table S1). Hence, the presence of genes with a known relevance in the context of T2D (24,25,45–48,51–53,140–152) provides a confirmation of the validity of the approach we used.

In addition to this, we found 25 genes with no previous association with T2D described in the literature. Among the criteria used to associate novel genes with a potential β-cell role, we considered the presence of interactions with known genes. ‘Synthetic lethals’ are gene pairs for which the deletion/inactivation of a single gene has no major phenotypic effects, whereas the inactivation of both genes produces a lethal phenotype. This kind of genetic interaction allowed us to link C19orf60 and SDR39U1 with MYC and RAS (52), respectively, which are relevant for β-cell differentiation, proliferation and apoptosis (51,53). We also found novel genes displaying proven functional interactions. For instance, DNAJA4 has been shown in human colon cancer cells to be regulated by SREBP and act as a mediator of lipotoxicity through ER stress (55). UBALD1 and OTUD3 are enzymes involved in ubiquitination with an experimentally validated interaction with MLYCD and PTEN, respectively, and potentially affecting β-cell metabolic pathways, function and turnover (153). The protein encoded by CKS1B binds SKP2, increasing the activity of the E3 ubiquitin ligase Skp1–Cullin-1–Skp2 that degrades p27 (50), a pathway shown to be involved in regulating β-cell mass and function with implications for T2D development (49). Other novel transcripts that we found to be differentially expressed in diabetic β-cells (such as the zinc finger protein ZC3H8 and CABIN1) are involved in mechanisms potentially associated with β-cell dysfunction, such as the regulation of intracellular calcium signaling (56,154,155). Therefore, our integrative analysis partly reconciled the previous fully inconsistent results reported with β-cell scRNA-seq.

We then assessed how the results of our meta-analysis compared with those from a relatively similar human dataset (microarray of laser capture microdissection of T2D and control islets) (26) and a less similar rat model (RNA-seq of whole islets following hyperglycemia induced by partial pancreatectomy) (27). Using the overlapping transcriptomic signature (RRHO) approach, we observed that there were several genes differentially expressed in the same direction in our results and those from MAR (60), where the authors used β-cell enriched preparation from ND and T2D donors. Interestingly, islets from hyperglycemic pancreatectomized rats (27) showed several changes in gene expression similar to those in our integrated human β-cell datasets. Furthermore, based on the analysis of genes differentially expressed (*P* ≤ 0.05) in the three datasets, we observed 49 shared genes (48 upregulated). Therefore, despite remarkable differences between the three models (single human β-cells yielded after islet digestion and separated by FACS or a microfluidic platform; β-cell enriched preparations obtained by LCM from the pancreas of organ donors; isolated islets from rats with surgically induced hyperglycemia; use of microarray or RNAseq), a set of shared DEGs remains associated with the dysfunctional β-cell across biological models and experimental techniques. These genes might represent key factors involved in the trajectory of β-cell failure (see Supplementary Table S4). For instance, upregulation of LDHA has a demonstrated involvement in perturbed insulin secretion (92), and overexpression of RPS10 is a marker of a functionally immature phenotype (93). Among the downregulated genes, ATP2A2 and PGRMC1 have a role in insulin secretion (127,131). Other shared genes without previously illustrated associations could provide novel insights into signatures of β-cell dysfunction. As an example, the gene PELP1 encodes a coactivator involved in a number of signaling pathways, including SRC/PI3K/AKT and ERK/MAPK, that can be relevant for defective insulin secretion (129,130).

In conclusion, this work represents the first integration of human islet single-cell transcriptomes to understand β-cell dysfunction in human T2D. The dataset we assembled (available at https://github.com/EBosi/scPanBetaT2D) has allowed to (i) partly reconcile the previously reported inconsistencies in single-cell analysis of human islet cells, (ii) identify novel genes to be investigated in future studies to better characterize the molecular basis of T2D onset and progression, and (iii) underscore specific traits of dysfunctional β-cells across different models and techniques.

## DATA AVAILABILITY

The ID numbers of the gene expression series used in this study are E-MTAB-5061 (14), SRP075377 (13), SRP075970 (15), GSE20966 (60) and GSE134966 (27).

The source Python and R code, the intermediary files and the h5ad file encoding the integrated dataset are available on https://github.com/EBosi/scPanBetaT2D.

## Supplementary Material

lqaa097_Supplemental_Files
