# Correction to ‘Integration of single-cell datasets reveals novel transcriptomic signatures of β-cells in human type 2 diabetes’

**DOI:** 10.1093/nargab/lqab053

**Published:** 2021-05-26

**Authors:** Emanuele Bosi, Lorella Marselli, Carmela De Luca, Mara Suleiman, Marta Tesi, Mark Ibberson, Decio L Eizirik, Miriam Cnop, Piero Marchetti

**Affiliations:** Department of Experimental and Clinical Medicine, Pancreatic Islets Laboratory, University of Pisa, Pisa, I-56124, Italy; Department of Experimental and Clinical Medicine, Pancreatic Islets Laboratory, University of Pisa, Pisa, I-56124, Italy; Department of Experimental and Clinical Medicine, Pancreatic Islets Laboratory, University of Pisa, Pisa, I-56124, Italy; Department of Experimental and Clinical Medicine, Pancreatic Islets Laboratory, University of Pisa, Pisa, I-56124, Italy; Department of Experimental and Clinical Medicine, Pancreatic Islets Laboratory, University of Pisa, Pisa, I-56124, Italy; Vital-IT Group, SIB Swiss Institute of Bioinformatics, University of Lausanne, Quartier Sorge, CH-1015 Lausanne, Switzerland; ULB Center for Diabetes Research, Université Libre de Bruxelles, Brussels, B-1070, Belgium; Indiana Biosciences Research Institute (IBRI), Indianapolis, IN 46202, USA; ULB Center for Diabetes Research, Université Libre de Bruxelles, Brussels, B-1070, Belgium; Division of Endocrinology, Erasmus Hospital, Université Libre de Bruxelles, Brussels, B-1070, Belgium; Department of Experimental and Clinical Medicine, Pancreatic Islets Laboratory, University of Pisa, Pisa, I-56124, Italy

The authors wish to correct the Funding statement of their article (1) to:
