# Supplementary material for: Integration of single-cell datasets reveals novel transcriptomic signatures of β-cells in human type 2 diabetes
Source: NAR Genom Bioinform. 2020 Nov 20;2(4):lqaa097. doi: 10.1093/nargab/lqaa097 (PMC7679065; doi:10.1093/nargab/lqaa097)
Supplement: lqaa097_Supplemental_Files [file lqaa097_supplemental_files.zip › Supplementary Data.docx]

# Supplementary Data

Supplementary File 1: Merged dataset provided as a h5ad file.

Supplementary Table 1: List of T2D DEGs in β-cells and pertinent references.

Supplementary Table 2: Functional enrichment results of T2D DEGs.

Supplementary Table 3: Annotated single β-cell DEGs overlapping with MAR and EBR according to RRHO, and pertinent references.

Supplementary Table 4: Annotated DEGs shared between single-cell, MAR and EBR, and pertinent references.

Supplementary Figure 1: Ranked cell-type specific genes.

Supplementary Figure 2: DEGs overlap between individual datasets
