# Supplementary material for: Integration of single-cell datasets reveals novel transcriptomic signatures of β-cells in human type 2 diabetes
Source: NAR Genom Bioinform. 2020 Nov 20;2(4):lqaa097. doi: 10.1093/nargab/lqaa097 (PMC7679065; doi:10.1093/nargab/lqaa097)
Supplement: lqaa097_Supplemental_Files [file lqaa097_supplemental_files.zip › Supplementary Figure 1.pdf]

Supplementary Figure 1

Panels of Marker Genes.

The different panels show for each cell type the top 20 genes that are most differentially expressed compared with the rest (Wilcoxon test). The x and y axes report, respectively, the rank values and the z-scores.

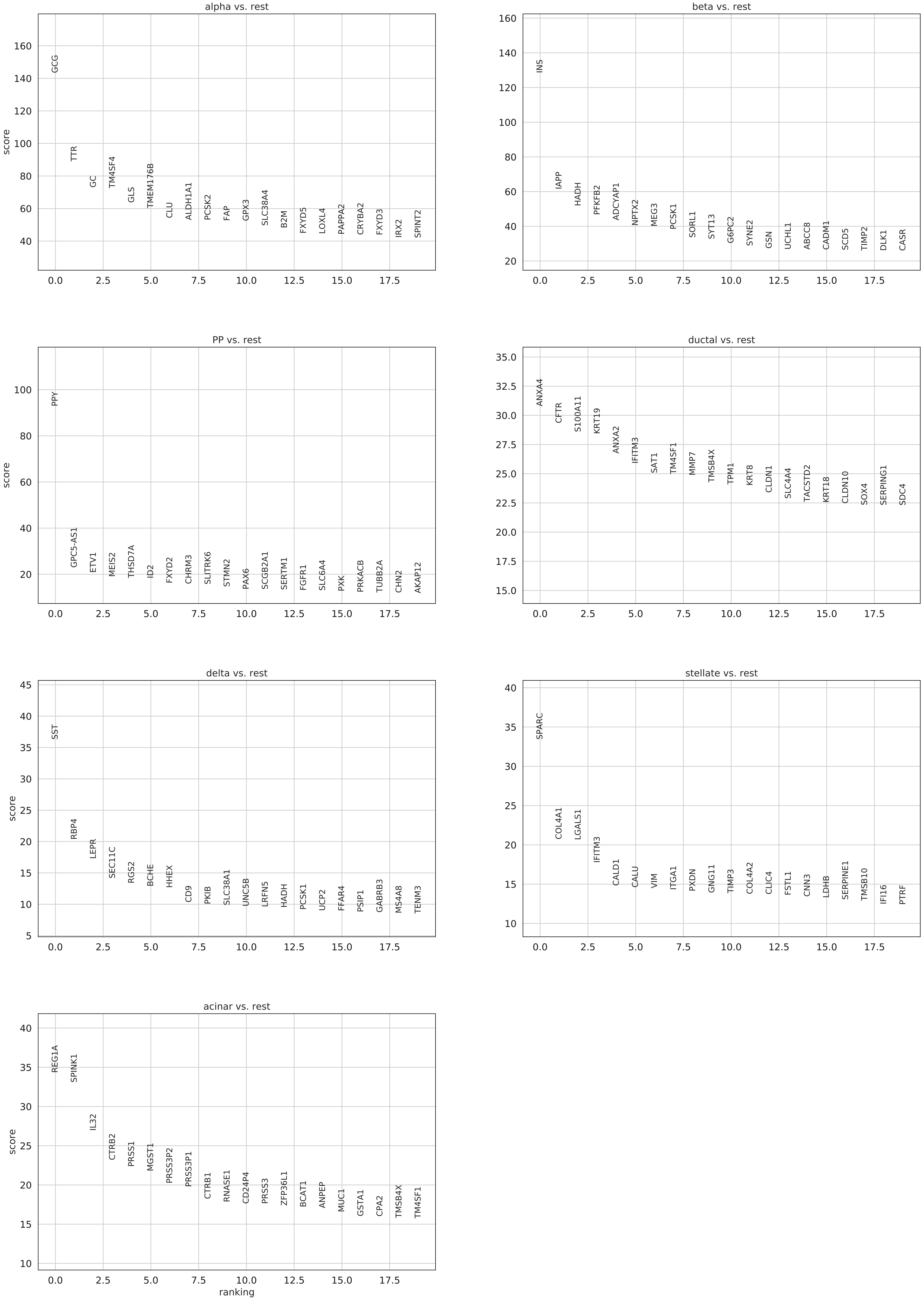
alpha vs. rest
score
GCG
TTR
GC
TM4SF4
GLS
TMEM176B
CLU
ALDH1A1
PCSK2
FAP
GPX3
SLC38A4
B2M
FXYD5
LOXL4
PAPPA2
CRYBA2
FXYD3
IRX2
SPINT2
beta vs. rest
INS
IAPP
HADH
PFKFB2
ADCYAP1
NPTX2
MEG3
PCSK1
SORL1
SYT13
G6PC2
SYNE2
GSN
UCHL1
ABCC8
CADM1
SCD5
TIMP2
DLK1
CASR
PP vs. rest
score
PPY
GPC5-AS1
ETV1
MEIS2
THSD7A
ID2
FXYD2
CHRM3
SLITRK6
STMN2
PAX6
SCGB2A1
SERTM1
FGFR1
SLC6A4
PXK
PRKACB
TUBB2A
CHN2
AKAP12
ductal vs. rest
ANXA4
CFTR
S100A11
KRT19
ANXA2
IFITM3
SAT1
TM4SF1
MMP7
TMSB4X
TPM1
KRT8
CLDN1
SLC4A4
TACSTD2
KRT18
CLDN10
SOX4
SERPING1
SDC4
delta vs. rest
score
SST
RBP4
LEPR
SEC11C
RGS2
BCHE
HHEX
CD9
PKIB
SLC38A1
UNC5B
LRFN5
HADH
PCSK1
UCP2
FFAR4
PSIP1
GABRB3
MS4A8
TENM3
stellate vs. rest
SPARC
COL4A1
LGALS1
IFITM3
CALD1
CALU
VIM
ITGA1
PXDN
GNG11
TIMP3
COL4A2
CLIC4
FSTL1
CNN3
LDHB
SERPINE1
TMSB10
IFI16
PTRF
acinar vs. rest
score
REG1A
SPINK1
IL32
CTRB2
PRSS1
MGST1
PRSS3P2
PRSS3P1
CTRB1
RNASE1
CD24P4
PRSS3
ZFP36L1
BCAT1
ANPEP
MUC1
GSTA1
CPA2
TMSB4X
TM4SF1
ranking
