# Supplementary figures and images for: Integration of single-cell datasets reveals novel transcriptomic signatures of β-cells in human type 2 diabetes
Source: NAR Genom Bioinform. 2020 Nov 20;2(4):lqaa097. doi: 10.1093/nargab/lqaa097 (PMC7679065; doi:10.1093/nargab/lqaa097)

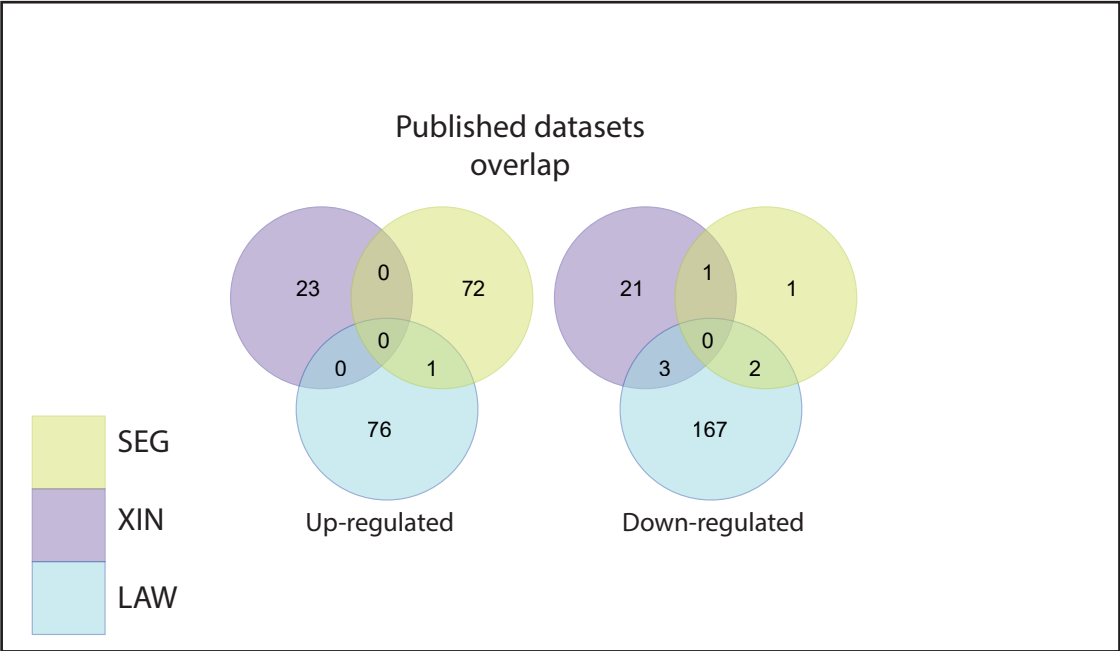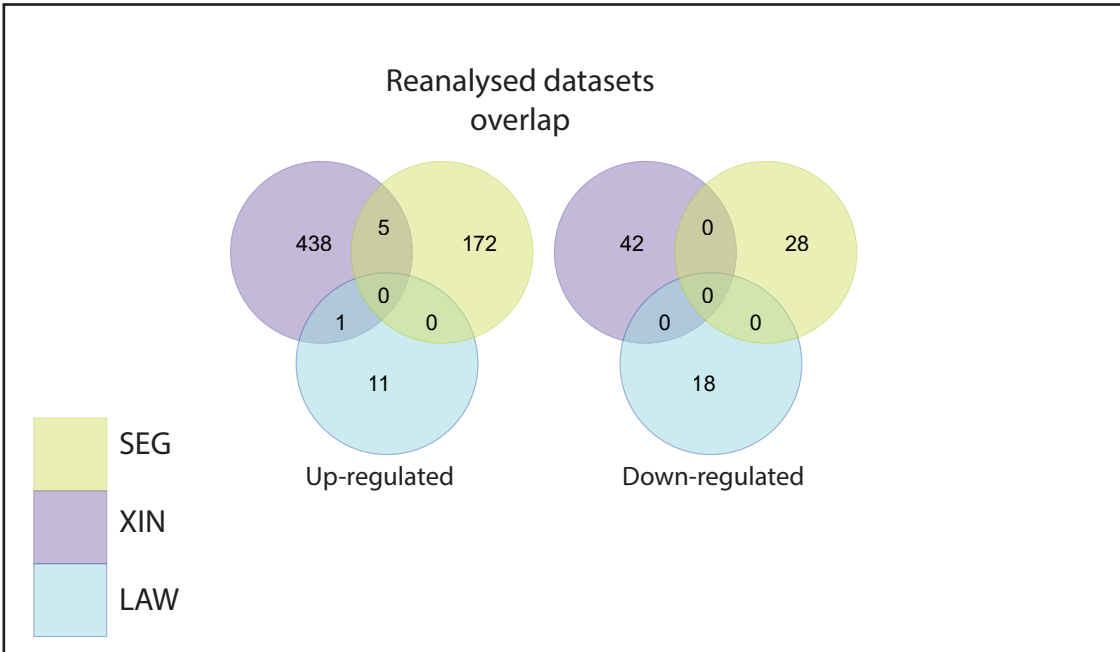

Supplement: lqaa097_Supplemental_Files [file lqaa097_supplemental_files.zip › Supplementary Figure 2.pdf]
